# Supplementary material for: Content-rich biological network constructed by mining PubMed abstracts
Source: BMC Bioinformatics. 2004 Oct 8;5:147. doi: 10.1186/1471-2105-5-147 (PMC528731; doi:10.1186/1471-2105-5-147)
Supplement: Additional File 2 — The original results of the above study (non-essential files are deleted to keep the file size under the limit set by BMC bioinformatics). [file 1471-2105-5-147-S2.bz2 › chilibotAdditionalFile2/dip50/445ID9468473E10451/html/CCL4_CCR5.html]

 


 **CCL4** and **CCR5** 
  
Found 58 abstracts in PubMed, retrieved 50.  
 

 What does Google say? 
 PDF only 
| .edu only 

---

**Interactive relationship** (e.g. stimulation, inhibition, etc)

**Stimulatory relationship**- The increased expression of  **CCR5**  resulted in enhanced chemotaxis toward the  **CCR5**  ligand macrophage inflammatory protein 1beta  **CCL4** , but up regulation of CCR6 did not result in altered chemotactic responsiveness to macrophage inflammatory protein 3alpha CCL20, suggesting an alternative function for this receptor.  Ref: 11751947 J Immunol, 2002
- Increased responsiveness of murine eosinophils to MIP 1beta  **CCL4**  and TCA 3 CCL1 is mediated by their specific receptors,  **CCR5**  and CCR8.  Ref: 12050188 J Leukoc Biol, 2002
- Stimulation of monocyte derived DCs with CD40LT enhanced the production of the CC chemokines macrophage inflammatory protein MIP 1 alpha, MIP 1 beta  [ **CCL4** ] , and RANTES and diminished surface expression of  **CCR5** .  Ref: 10092834 J Immunol, 1999
- The higher RANTES production by MS gamma delta T cell lines, together with a lower expression of  **CCR5** , may reflect an autoregulatory loop, caused by an increased production of its ligands RANTES, MIP 1 alpha, and MIP 1 beta  [ **CCL4** ]  or due to other pro inflammatory cytokines.  Ref: 12173306 Clin Immunol, 2002
- A major advance in understanding human immunodeficiency virus HIV biology was the discovery that the beta chemokines MIP 1 alpha macrophage inflammatory protein 1 alpha, MIP 1 beta  [ **CCL4** ]  macrophage inflammatory protein 1 beta  [ **CCL4** ]  and RANTES regulated on activation, normal T cell expressed and secreted inhibit entry of HIV 1 into CD4 cells by blocking the critical interaction between the  **CCR5**  coreceptor and the V3 domain of the viral envelope glycoprotein gp120.  Ref: 9512422 Curr Biol, 1998
**Neutral relationship**- no modulation of  **CCR5**  expression was observed, nor was MIP 1 beta  [ **CCL4** ]  binding to  **CCR5**  altered.  Ref: 10477578 J Immunol, 1999
- Chemokines, including IP 10, Mig, Mip 1 alpha, and Mip 1 beta  [ **CCL4** ] , may participate in the selective recruitment of  **CCR5**  CXCR3 T cells to the inflamed synovium.  Ref: 11141325 Clin Immunol, 2001
- Moreover, cells stained with fluorescently labeled MIP 1 beta  [ **CCL4** ]  extensively colocalized with the GM1 lipid raft marker while using anti  **CCR5**  antibodies.  Ref: 12036855 Blood, 2002
- However, the remaining expression of CCR1 and  **CCR5**  correlated reciprocally with an ongoing unresponsiveness to the CCR1 and  **CCR5**  agonists CCL3 macrophage inflammatory protein 1alpha MIP 1alpha,  **CCL4**  MIP 1beta, and CCL5 regulated on activation, normal T expressed and secreted RANTES, all chemokines binding to these two receptors.  Ref: 12885942 J Leukoc Biol, 2003
- Reciprocal desensitization of  **CCR5**  and CD4 is mediated by IL 16 and macrophage inflammatory protein 1 beta  [ **CCL4** ] , respectively.  Ref: 10477578 J Immunol, 1999
- A number of inflammatory CC chemokines, including MIP 1 alpha, MIP 1 beta  [ **CCL4** ] , RANTES, MCP 2, and HCC 1 9 74 act as  **CCR5**  agonists, while MCP 3 is a natural antagonist of the receptor.  Ref: 12402506 Receptors Channels, 2002
- High levels of CCL3 and  **CCL4**  were produced within 8 h after infection, which is likely to account for the observed  **CCR5**  down modulation on Mtb infected DC.  Ref: 12538673 J Immunol, 2003
- Importance of basic residues and quaternary structure in the function of MIP 1 beta  [ **CCL4** ]   **CCR5**  binding and cell surface sugar interactions.  Ref: 11305915 Biochemistry, 2001
- **CCR5**  is a G protein coupled receptor responding to four natural agonists, the chemokines RANTES regulated on activation normal T cell expressed and secreted, macrophage inflammatory protein MIP 1 alpha, MIP 1 beta  [ **CCL4** ] , and monocyte chemotactic protein MCP 2, and is the main co receptor for the macrophage tropic human immunodeficiency virus strains.  Ref: 12411445 J Biol Chem, 2003
- In human monocytes pretreatment with eotaxin decreased responsiveness to MCP 1, a selective ligand for CCR2, as well as to RANTES and MIP 1 beta  [ **CCL4** ] , which bind to  **CCR5** .  Ref: 11264152 Blood, 2001
- Functional studies of the purified protein revealed that MIP 1 beta  [ **CCL4** ]  3 69 retains the abilities to induce down modulation of surface expression of the chemokine receptor  **CCR5**  and to inhibit the  **CCR5**  mediated entry of HIV 1 in T cells.  Ref: 12070155 J Biol Chem, 2002
- Several potential mechanisms for expansion of HIV 1 in situ were found, including augmentation in expression of tumor necrosis factor TNF alpha and the HIV 1 noninhibitory beta chemokine MCP 1, low presence of HIV 1 inhibitory beta chemokines MIP 1 alpha, MIP 1 beta  [ **CCL4** ] , and RANTES regulated on activation, normal T expressed and secreted, and upregulation in expression of the HIV 1 coreceptor,  **CCR5** , by pleural fluid mononuclear cells.  Ref: 11579270 J Acquir Immune Defic Syndr, 2001

**Non-interactive relationship** (e.g. studied together, co-existance, homology, etc.)

- We found that cholesterol extraction by hydroxypropyl beta cyclodextrin BCD significantly reduced the binding and signaling of macrophage inflammatory protein 1 beta  [ **CCL4** ]  MIP 1 beta  [ **CCL4** ]  using  **CCR5**  expressing CEM NKR T cells.  Ref: 12036855 Blood, 2002
- We analyzed sera from ESN, their HIV infected sexual partners HIV, and healthy controls USN searching for  **CCR5**  specific Abs, studying whether incubation of PBMC with sera could prevent macrophage inflammatory protein 1 beta  [ **CCL4** ]  Mip1 beta natural ligand of  **CCR5**  binding to  **CCR5** .  Ref: 10706739 J Immunol, 2000
- The most active chemokines for resting Th IL 10, in terms of calcium mobilization and in vitro migration, were in order of potency CCL2 monocyte chemoattractant protein 1, CCR2 ligand,  **CCL4**  macrophage inflammatory protein 1beta,  **CCR5**  ligand, CCL3 macrophage inflammatory protein 1alpha, CCR1 5 ligand, CCL17 thymus and activation regulated chemokine, CCR4 ligand, CCL1 I 309, CCR8 ligand, CXCL12 stromal derived factor 1, CXCR4, and CCL11 eotaxin, CCR3 ligand.  Ref: 11145678 J Immunol, 2001
- We examined the responses of human peripheral blood T cells, present in an ECM like context, to combinatorial signaling transduced by SDF 1alpha CXCL12, and two  **CCR5**  ligands, RANTES CCL5 and MIP 1beta  **CCL4** .  Ref: 12502723 Int Immunol, 2003
- In this study, the expression of CCR3,  **CCR5** , CXCR3, and CXCR4 were analyzed by flow cytometry in three distinct in vitro models of Th1 Th2 polarization, activated naive and memory T cells, and T cell clones, in which the intracellular synthesis of interferon gamma IFN gamma and interleukin 4 IL 4 and the surface expression of CD30 and LAG  [ **CCL4** ]  3 were also assessed.  Ref: 10331500 J Leukoc Biol, 1999
- These data suggest that the MIP 1 beta  [ **CCL4** ]  dimer is not required for  **CCR5**  binding or activation.  Ref: 10727234 Biochemistry, 2000
- The effect is not unique to CCL5, as other  **CCR5**  ligands such as CCL3 MIP 1alpha and  **CCL4**  MIP 1beta also trigger TIL cell death, nor is it limited to melanoma cells, as it also operates in activated primary T lymphocytes.  Ref: 11369232 Curr Biol, 2001
- After 2 to 4 weeks, L. amazonensis infected mice had significantly delayed and depressed expression of inflammatory cytokines interleukin 12 IL 12, gamma interferon, IL 1alpha, IL 1beta, CC chemokines CC chemokine ligand 3 CCL3 macrophage inflammatory protein 1alpha MIP 1alpha,  **CCL4**  MIP 1beta, CCL5 RANTES, MIP 2, and chemokine receptors CCR1, CCR2,  **CCR5**  in foot tissues and draining lymph nodes compared to the expression in L. major infected controls.  Ref: 12874303 Infect Immun, 2003
- Characterization of the role of the N loop of MIP 1 beta  [ **CCL4** ]  in  **CCR5**  binding.  Ref: 12427015 Biochemistry, 2002
- This effect was partially blocked by anti MIP 1 beta  [ **CCL4** ]  and anti CC chemokine receptor 5  [ **CCR5** ]  Ab, but not by anti MIP 1 alpha Ab suggesting that MIP 1 beta  [ **CCL4** ]  plays a major role in this chemoattraction.  Ref: 10201982 J Immunol, 1999
- These studies demonstrate that IL 16 CD4 signaling in T lymphocytes results in a selective loss of macrophage inflammatory protein MIP 1 beta  [ **CCL4** ]   **CCR5**  induced chemotaxis.  Ref: 10477578 J Immunol, 1999
- In LAG  [ **CCL4** ]  3 matured DC, surface expression of  **CCR5**  was down regulated and CCR7 was up regulated.  Ref: 12547595 Vaccine, 2003
- In addition, we identified Phe13, the residue immediately following the conserved CC motif in MIP 1 beta  [ **CCL4** ] , as a key determinant for binding to  **CCR5** .  Ref: 10727234 Biochemistry, 2000
- Characterization of the chemokine receptor specificity of MIP 1 beta  [ **CCL4** ]  3 69 showed that the truncated protein not only shares the ability of intact MIP 1 beta  [ **CCL4** ]  to induce Ca 2 signaling through  **CCR5** , but unlike the full length protein, it also triggers a Ca 2 response via CCR1 and CCR2b.  Ref: 12070155 J Biol Chem, 2002
- Sulfated tyrosines contribute to the binding of  **CCR5**  to MIP 1 alpha, MIP 1 beta  [ **CCL4** ] , and HIV 1 gp120 CD4 complexes and to the ability of HIV 1 to enter cells expressing  **CCR5**  and CD4.  Ref: 10089882 Cell, 1999
- Functional relevance was determined by receptor binding affinity and the ability to invoke intracellular calcium release from CHO cells transfected with the MIP 1 beta  [ **CCL4** ]  receptor  **CCR5** .  Ref: 10727234 Biochemistry, 2000
- **CCR5**  and LAG  [ **CCL4** ]  3 exhibited the same pathway of Th1 association.  Ref: 10331500 J Leukoc Biol, 1999
- We also determined that  **CCR5**  binding chemokines MIP 1alpha CCL3,  **CCL4** , and RANTES CCL5, specifically by signaling through  **CCR5** , could affect all progenitor B cell populations through a novel mechanism involving heterologous desensitization of CXCR4.  Ref: 12239139 Blood, 2002
- Th1 related molecules include CD26, membrane IFN gamma, LAG  [ **CCL4** ]  3,  **CCR5**  and CXCR3.  Ref: 9831180 Eur Cytokine Netw, 1998
- Cholesterol is essential for macrophage inflammatory protein 1 beta  [ **CCL4** ]  binding and conformational integrity of CC chemokine receptor 5  [ **CCR5** ] .  Ref: 12036855 Blood, 2002
- In order to evaluate if these cells are infectabile by human immunodeficiency virus HIV and could be employed as a model in HIV research we exposed these cell lines to X4 T cell tropic and R5 macrophage tropic and subsequently tried to correlate their infectability with i level of chemokine coreceptor CXCR4 and  **CCR5**  expression, ii coreceptor functionality calcium flux, chemotaxis and phosphorylation of MAPK p42 44 and AKT and iii endogenous expression and secretion of HIV related chemokines which compete with the virus for binding to CXCR4 SDF 1 CXCL12 or  **CCR5**  MIP 1beta  **CCL4** , MIP 1alpha CCL3, RANTES CCL5, MCP 2 CCL8, MCP 3 CCL7 and MCP 4 CCL13.  Ref: 11737246 Eur J Haematol, 2001
- The beta chemokines, macrophage inflammatory protein MIP 1 alpha, MIP 1 beta  [ **CCL4** ] , monocyte chemotactic protein MCP 1 and regulated on activation normal T cell, expressed and secreted RANTES are not only chemotactic for mononuclear cells but may be important in suppression of HIV 1 replication through competitive binding to the chemokine receptor,  **CCR5** , which is critical to viral entry.  Ref: 11317070 J Acquir Immune Defic Syndr, 2001
